# Rapid and efficient immunomagnetic isolation of endothelial cells from human peripheral nerves

**DOI:** 10.1038/s41598-021-81361-x

**Published:** 2021-01-21

**Authors:** Patrick Dömer, Janine Kayal, Ulrike Janssen-Bienhold, Bettina Kewitz, Thomas Kretschmer, Christian Heinen

**Affiliations:** 1 Department of Neuroscience, Carl Von Ossietzky University Oldenburg, Carl von Ossietzky Str. 9-11, Oldenburg, Germany; 2 Department of Neurosurgery, Evangelisches Krankenhaus, Campus Carl von Ossietzky University Oldenburg, Oldenburg, Germany; 3 Research Center Neurosensory Science, Carl Von Ossietzky University Oldenburg, Oldenburg, Germany; 4 Department of Neurosurgery and Neurorestauration, Klinikum Klagenfurt Am Wörthersee, Klagenfurt, Austria

**Keywords:** Peripheral nervous system, Regeneration and repair in the nervous system

## Abstract

Endothelial cells (ECs) have gained an increased scientific focus since they were reported to provide guidance for Schwann cells and subsequently following axons after nerve injuries. However, previous protocols for the isolation of nerve-derived ECs from human nerves are ineffective regarding time and yield. Therefore, we established a novel and efficient protocol for the isolation of ECs from human peripheral nerves by means of immunomagnetic CD31-antibody conjugated Dynabeads and assessed the purity of the isolated cells. The easy-to-follow and time-effective isolation method allows the isolation of > 95% pure ECs. The isolated ECs were shown to express highly specific EC marker proteins and revealed functional properties by formation of CD31 and VE-cadherin positive adherens junctions, as well as ZO-1 positive tight-junctions. Moreover, the formation of capillary EC-tubes was observed in-vitro. The novel protocol for the isolation of human nerve-derived ECs allows and simplifies the usage of ECs in research of the human blood-nerve-barrier and peripheral nerve regeneration. Additionally, a potential experimental application of patient-derived nerve ECs in the in-vitro vascularization of artificial nerve grafts is feasible.

## Introduction

The peripheral nerve’s vascularization is composed of blood vessels of different size and physiological function. Fenestrated macrovessels (the *vasa nervorum*) are located within the epineurium, while exclusively non-fenestrated microvessels were found within the nerve fascicles^1,2^. In addition to their primary function, the supply with oxygen and nutrients, several other physiological roles of the nerve-vasculature have been uncovered. In particular, the intrafascicular microvascular endothelial cells (EC) have been identified as important components involved in the formation and maintenance of the blood nerve barrier (BNB)^3,4^. Herein, the microvascular non-fenestrated ECs form a selectively permeable barrier between the bloodstream and the endoneurium, while the perineurial cells seal the fascicle from the outside via tight junctions^2,4^. These barriers allow the control and maintenance of the endoneurial homeostasis, which is vital for functional signaling of peripheral nerves^2–4^. Furthermore, based on nerve transection studies, Cattin et al. provided evidence that neural microvessels also play an important role in axonal guidance by mediating Schwann cell migration along endothelial cells^5^. Taken together these findings point to the high scientific relevance of nerve-derived ECs for research on the BNB and peripheral nerve regeneration. Nevertheless, until today cell culture studies involving ECs are mainly based on the commercially available human umbilical vein endothelial cells (HUVECs)^6,7^ or immortalized endothelial cell lines^8–10^, despite a major disadvantage. As uncovered by more detailed studies, all of these cell lines exhibit different protein expression patterns and physiological properties^8,11^ and even endothelial cells originating from different tissues differ significantly^8,12–15^. This holds also true for nerve-derived ECs, where different physiological properties and protein expression profiles have been described for ECs derived from epineurial macrovessels and endoneurial microvessels, respectively^15–17^. One major physiological difference is the formation of tight junctions between ECs of the intrafascicular BNB-forming microvessels^4,18,19^, which is highly reduced in the macrovasculature^4,18,20^. For this reason, we believe that primary human ECs derived from the endoneurial compartment of peripheral nerves should be used for further research. This is particularly important for studies regarding the distinctive functions of ECs and their potential to promote regeneration in human peripheral nerves. Unfortunately, a protocol for a fast, easy-to-follow and highly efficient isolation procedure generating nearly pure primary endothelial cells from human nerves is still missing. Previous protocols used time-consuming density gradient centrifugation^9,15,21^, a method which not only requires significant experience in density centrifugation to prevent contamination, but also needs expensive equipment and is work intensive.

To overcome these methodological deficits, we established a simple, time-efficient and rather inexpensive isolation protocol for ECs from human sural nerves by means of immunomagnetic cell sorting using CD31 coated Dynabeads.

## Results

### Cell viability and anti-CD31-Dynabead conjugation

**Figure 1:**
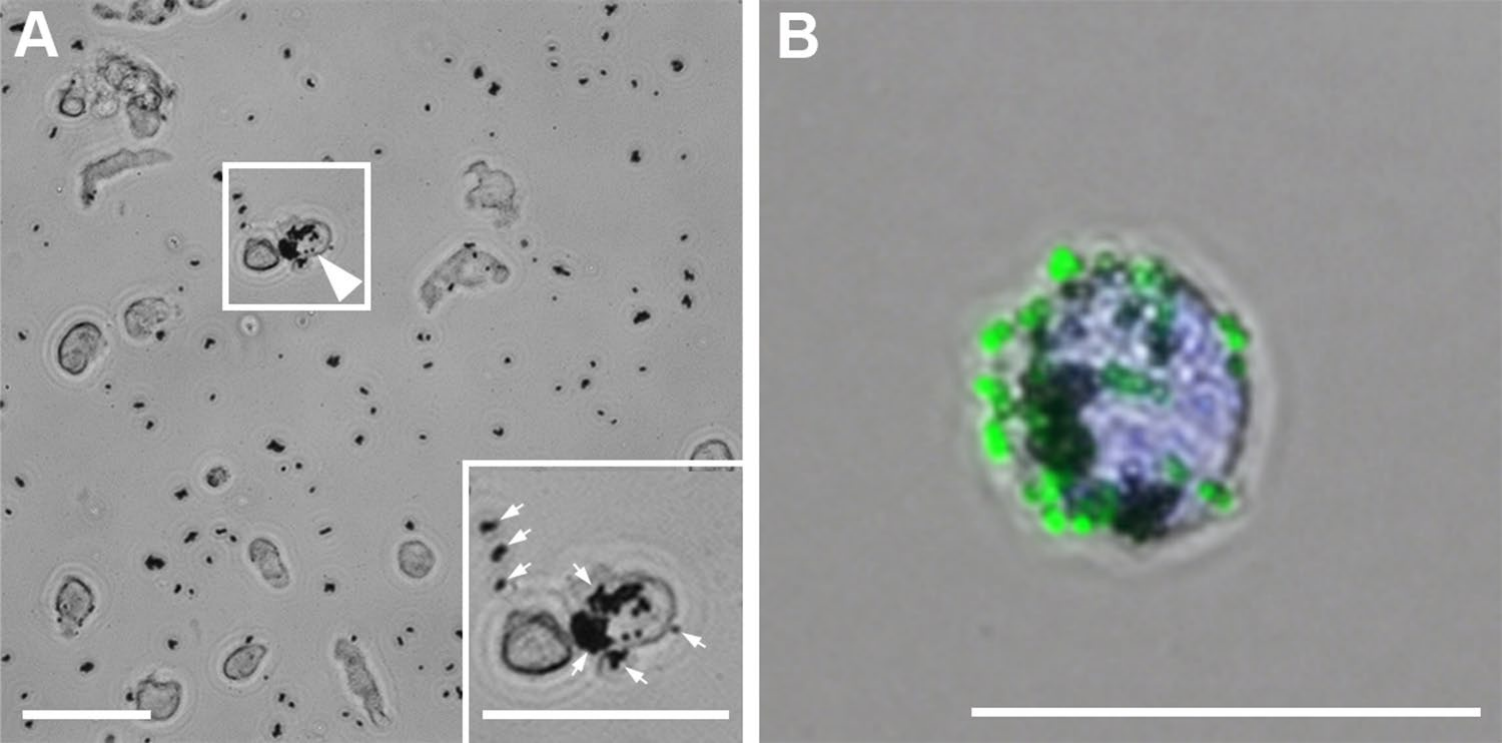
Dynabeads conjugated with anti-CD31 show binding to endothelial cells (white arrowhead and inset), whereas other non-endothelial cells were free of Dynabeads (**A**). Unbound and cell-attached Dynabeads can be observed as black dots (inset, small arrows). For verification of the anti-CD31-Dynabead conjugation, the ECs with their bound anti-CD31 Dynabeads were incubated with Alexa488 conjugated secondary antibodies directed against mouse IgGs, detecting the primary anti-CD31 antibody conjugated to the Dynabeads (**B**). The entire amount of anti-CD31 antibody was conjugated to the Dynabeads, since no CD31 staining of the EC membrane was detected. Scale: 50 µm.

For an optimal isolation of highly enriched endothelial cells from human nerve, the nerve tissue must be properly digested to obtain a single cell solution. This is an essential step before the subsequent immunomagnetic enrichment with CD31-antibody coated Dynabeads can be carried out. Digestion with collagenase and hyaluronidase for > 12 h and follow-up trituration resulted in a single cell suspension (see Fig. 1A) and a cell viability of > 90%, as assessed by the trypan-blue assay. To ensure a reliable interaction of the magnetic Dynabeads with endothelial cells, a sufficient anti-CD31-Dynabead conjugation is a decisive prerequisite. To prove this conjunction, Alexa488 conjugated anti-mouse IgG was used to control for proper binding of the anti-CD31-antibody to the Dynabeads as well as the binding of the Dynabeads to ECs. As shown in Fig. 1B, the anti-CD31 carrying Dynabeads showed an intense fluorescence signal while no fluorescence of the EC membrane was detected. This confirms a successful conjunction of the anti-CD31 antibody and the Dynabeads and verifies the binding of the anti-CD31 Dynabeads to ECs. The absent staining of the EC membrane attests no unbound anti-CD31 antibody within the Dynabead suspension (see Fig. 1B).

### Isolation and culture of endothelial cells of the human nerve

**Figure 2:**
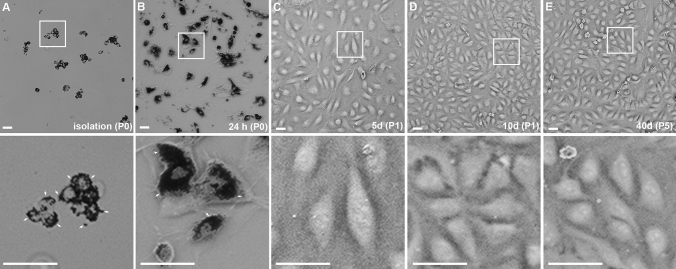
Isolation of endothelial cells with CD31 conjugated Dynabeads (**A**) and subsequent cultivation for 24 h, 5 days and 10 days. (**B**) 24 h after seeding, the cells with the bound Dynabeads (black dots, small arrows) have attached to the cell culture plate. (**C**) Following the first passaging 3 days after seeding, most of the Dynabeads were detached due to the trypsinization-process. At day 5, the endothelial cells proliferated (**C**) and showed vortical cell alignment when reaching confluency after 10 days (**D**). Following 5 passages, the proliferation rate declined but the ECs showed no signs of senescence (**E**). These results were reproduced four times with ECs derived from four independent isolation procedures of individual patients. Scale: 50 µm.

Following the digestion process, endothelial cells showed a small soma size with a spherical shape (see Fig. 2A). Aggregates of endothelial cells were observed frequently after immunomagnetic retention, probably due to cell attachment during the retention process or incomplete digestion due to the tight cell–cell junctions within the blood vessels (see Fig. 2A). However, the aggregates consisted predominantly of ECs and thus, a reduction of the aggregates was not pursued. Each endothelial cell was covered with multiple Dynabeads (black dots, see Figs. 1 and 2A,B; small arrows), the attachment of the ECs to the surface of the cell culture plate was however not constrained. Within 24 h of incubation, the ECs were attached and showed a bi- to multipolar morphology (see Fig. 2B). Dead cells as well as loosely- or unattached Dynabeads were removed by media change 24 h post-seeding and after reaching 90% confluence at day 3, splitting of the cells resulted in removal of Dynabeads attached to the ECs due to the trypsinization process. After 5 days, the ECs exhibited a bi- or tripolar elongated shape, without prominent cell alignment (see Fig. 2C). In contrast, at confluency (see Fig. 2D), the ECs possessed a triangular shape and formed vortical cell clusters, characteristic for endothelial cells. At this time point, the human nerve-derived ECs were passaged in a 3:1 ratio and could be used for experiments for up to 5 passages until the proliferation-rate declined. No signs of senescence were observed in up to 5 passages (see Fig. 2E). For storage, cryopreservation of isolated ECs directly after the isolation procedure indicated a recovery-rate of up to 95%. The cryopreservation of whole sural nerve samples prior to the isolation process led to non-viable cells, probably due to a lack of penetration of the freezing media.

### Molecular and immunocytochemical assessment of isolated ECs

**Figure 3:**
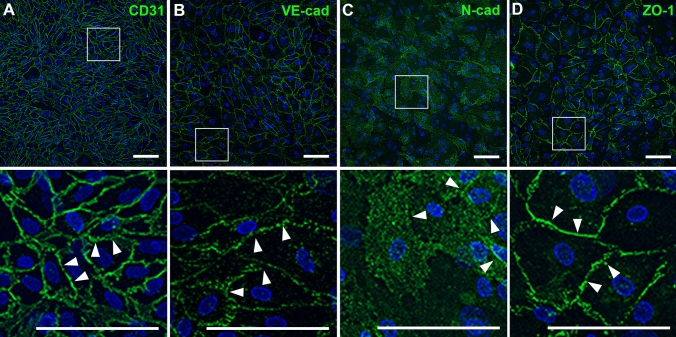
Immunocytochemical staining of endothelial cell marker proteins verified the endothelial origin of the ECs. Immunoreactivity against CD31 (**A**) as well as VE-cadherin (**B**) was detected at cell–cell contacts (arrowheads), while N-cadherin (**C**) showed a diffuse somatic staining of varying degree with weak accumulations at junctional contacts (arrowheads). The ZO-1 immunoreactivity (**D**) was found at cell–cell contacts labeling cellular junctions (arrowheads). These results were reproduced four times with ECs at passage 1 derived from four independent isolation procedures of individual patients. Scale: 100 µm.

For verification of the endothelial origin of the cultivated cells, the endothelial cell marker proteins CD31, VE-cadherin, the tight junction associated protein ZO1, as well as N-cadherin were immunocytochemically labeled. The peripheral nerve derived ECs showed an intense staining for the endothelial cell marker proteins CD31 and VE-cadherin, particularly at the contact sites between ECs (Fig. 3A,B, arrowheads). The evaluation of the EC purity by means of CD31 immunocytochemistry revealed a portion of > 95% positive cells. The immunoreactivity for N-cadherin was detected within the soma of endothelial cells, although the expression level varied between the ECs (Fig. 3C). Moreover, accumulations of N-cadherin were found in the membrane at cellular contact sites (arrowheads). Labeling of the tight junction associated protein ZO-1 showed an intense signal at the contact sites of ECs, suggesting the formation of tight-junction contacts between ECs (Fig. 3D, arrowheads).

**Figure 4:**
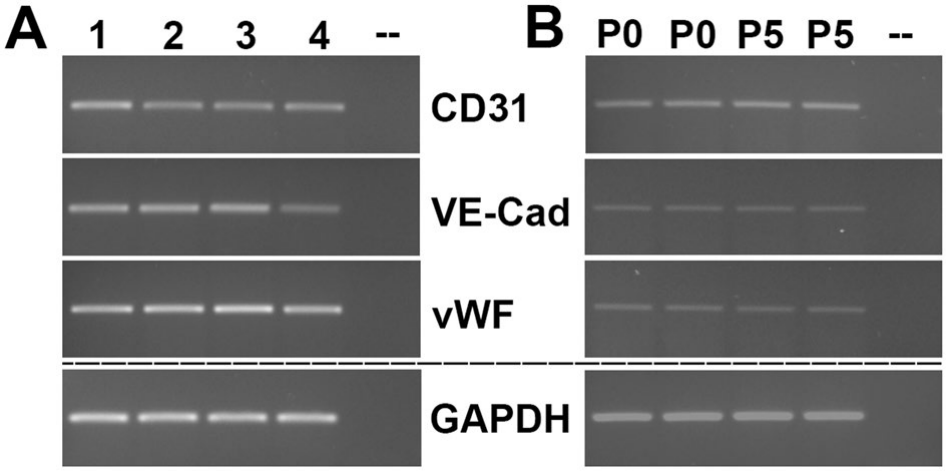
mRNA-Expression of the endothelial cell specific markers CD31, VE-Cadherin and von Willebrand factor was confirmed via RT-PCR in four independent EC isolations at passage 1 (1–4) (**A**). No differences were found in the expression patterns of CD31, VE-Cadherin, vWF and GAPDH between ECs gained directly after isolation (P0) and at passage 5 (**B**). Equal amounts of cDNA were used as verified by the GAPDH positive control. Omission of the template cDNA (–) revealed no signal. The PCR products of CD31, VE-Cadherin and von Willebrand factor were separated on the same gel, while GAPDH was separated independently. Uncropped gels can be found in Supplementary Fig. S1 in the Supplementary information file.

Using PCR, the expression of CD31, VE-cadherin and vWF was confirmed in four independent cell isolation procedures, each with nerve samples of different patients (Fig. 4A). While the amount of CD31 and VE-cadherin mRNA differed slightly, vWF was expressed analogously in all preparations compared to the load-control GAPDH. To analyze whether gene expression is affected by the number of passages, we compared the gene expression of CD31, VE-cadherin, vWF and GAPDH in non-passaged ECs and ECs of passage 5. However, no differences in gene expression profiles were observed (see Fig. 4B).

**Figure 5:**
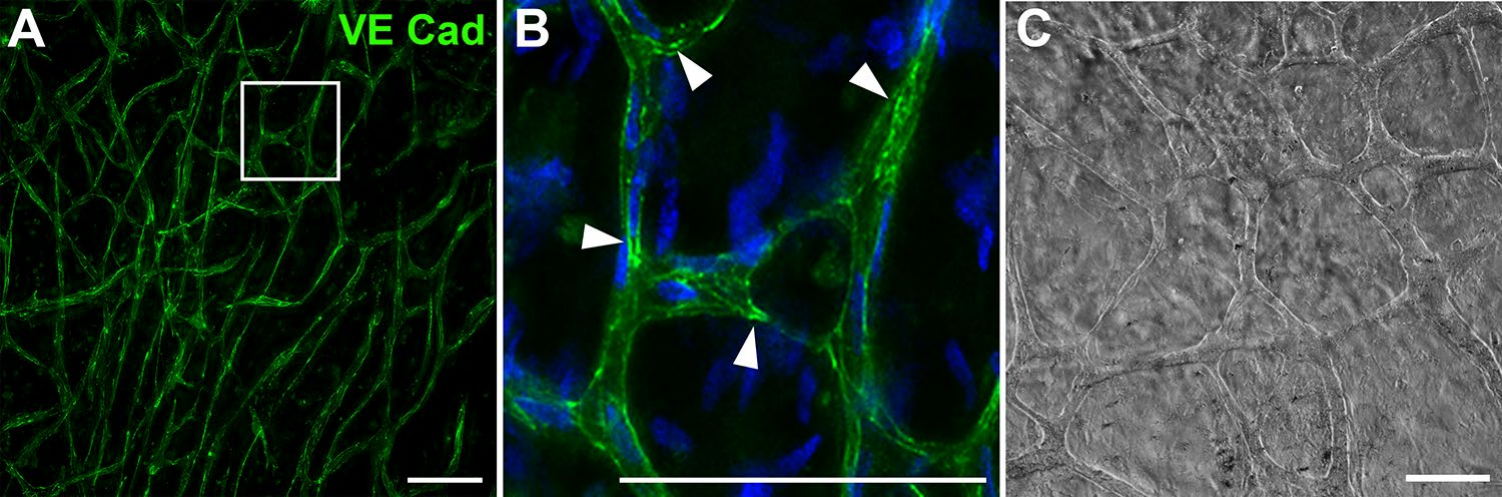
Tube formation of peripheral nerve derived ECs following cultivation on Matrigel under serum deprivation for 12 h. (**A**) Tube-forming endothelial cells, labeled by anti-VE-cadherin, revealed an intense immunoreactivity at endothelial adherens junctions (arrowheads) within the capillary tubes (**B**). Cell nuclei in the inset are labeled with DAPI (blue). (**C**) Brightfield image of EC tubes in regular media. These results were reproduced four times with ECs at passage 1 derived from four independent isolation procedures of individual patients. Scale: 100 µm.

The functional capabilities of peripheral nerve endothelial cells were verified either by cultivation on Matrigel under serum deprivation for 12 h (see Fig. 5) or by cultivation under regular conditions for one week after confluency had been reached. Both techniques resulted in the formation of endothelial tubes with a capillary-like structure (Fig. 5). These appear similar to endothelial tubes formed by HUVECs in in-vitro assays, which are frequently used to analyze the characteristic features of ECs and to test the angiogenic properties of distinct substances. The capillary network formed by peripheral nerve derived ECs is widely ramified, and the ECs exhibit functional adherens junctions, as confirmed by staining for VE-cadherin (see Fig. 5).

## Discussion

Peripheral nerve ECs have gained an increased scientific focus since they were proven indispensable for the maintenance of intrafascicular homeostasis due to their vital function within the nerve barrier^3,4^ and their important role in the mediation of axonal guidance during nerve regeneration^5,7^. But, the so far presented results require validation in the human system, since human nerve physiology and regenerative capacity was shown to differ significantly when compared to rodent model systems^22,23^. The most common cell culture model system for human ECs are primary HUVECs. Surprisingly, this specific EC cell type was even used in peripheral nerve research^5,7^, although the intrafascicular microvasculature differs in its physiological properties from the macrovascular origin of umbilical vein ECs. This physiological differences are underlined by a lack of continuous membrane associated ZO-1 in HUVECS^24,25^ as well as by a higher transendothelial resistance (TEER) of intrafascicular endothelial cells compared to HUVECS^18^. However, HUVECs are widely commercially available, whereas the isolation of ECs was previously associated with a costly and time-consuming isolation procedure. Thus, in the present study we aimed to establish a novel protocol for a more rapid and efficient procedure to isolate ECs and were successful in reaching a yield of > 95% pure peripheral nerve-derived ECs from human sural nerve samples by means of immunomagnetic anti-CD31 coated Dynabeads.

Due to the great interest in human nerve-derived micro- and macrovascular cells, other authors have reported procedures for the isolation of ECs from human peripheral nerves^9,15,21^. However, these protocols are time consuming and require vast experience in dextran or Percoll gradient centrifugation. Therefore, we established an easy-to-use isolation protocol for nerve derived ECs, based on immunomagnetic Dynabeads. The Dynabeads used in this protocol were streptavidin-coated, providing a low rate of false-positive cell isolation and a high variability, based on the vast choice of primary and secondary antibodies, which can be conjugated, only limited by the requirement of a well-fitting extracellular target-epitope^26^. For the isolation procedure, we decided to use an antibody directed against the CD31 epitope, since it has a large extracellular domain^27^ and has been proven highly specific for the immunocytochemical detection of ECs and their isolation from other tissues^27–30^.

Depending on the preparation of single fascicles prior to digestion, or the use of the whole nerve, either pure endoneurial microvascular cells or epineurial macro- and endoneurial microvascular cells can be isolated. However, the yield is substantially reduced using isolated fascicles. While the removal of adipose and connective tissue for whole nerve preparations can be performed without extensive training at low magnifications, the preparation of single fascicles requires a certain level of expertise and higher magnification equipment. Regarding tissue digestion, the incubation time strongly depends on the used enzymes and their concentration. While other protocols used a rapid digestion with higher enzyme concentrations mainly for soft tissue^28–31^, the solid cellular structure of peripheral nerves required, in our experience, a mild but prolonged digestion with collagenase I and hyaluronidase to achieve a single cell solution with a high cell-viability. During the subsequent isolation procedure, 5 µl CD31 conjugated Dynabeads per 5 cm nerve length have been proven adequate to gain the highest yields of viable ECs and without excess of unbound beads. One further essential step during the isolation procedure is the alignment of the bead-bound cells to the walls of the reaction tube, when placed inside the DynaMag-2 magnet. Therefore, the 5 min of incubation time within the magnet should not be reduced, as shorter times will lead to a significant reduction in the yield of ECs. For culturing of nerve-derived ECs, seeding densities should not be less than 2000 cells/cm^2^, because otherwise proliferation rates will be too low, since cell–cell contacts affect the proliferation rate, as it has also been observed in other studies^32^. A further advantage of the new EC- isolation protocol is, that a second purification step can be introduced before subculturing when the cells reach confluency, by using Dynabeads conjugated with another EC specific antibody, e.g. against the intercellular adhesion molecule 2^30^. However, the purity of CD31 positive cells was proven to be sufficient with a yield > 95%, and thus, the second purification step was omitted in this protocol. Although other protocols reported a purity of up to 98.9% for mouse endothelial cells^28^, a purity of 95% achieved by our protocol is comparable to the results received by Yosef et al. for human nerve derived ECs^15^.

Following the isolation procedure, a reduction in the number of cell-attached Dynabeads can be achieved by an exchange of the culture medium. However, following the trypsinization during subculturing, the Dynabeads were completely removed. This phenomenon can be traced to CD31 epitope-cleavage, since 63 trypsin cleavage sites are present in the 574 amino acids encompassing extracellular domain of the human CD31 protein (human CD31/PECAM1 (UniProt accession P16284; extracellular domain aa 28-601), Expasy PeptideCutter https://web.expasy.org/peptide_cutter/).

Within 24 h post isolation, the ECs exhibited the “cobblestone”-morphology, which is typical for cells of endothelial origin^33,34^. Despite these morphologic characteristics we observed in cultured ECs, several EC marker proteins were assessed via PCR and immunocytology. Immunolabeling of the purified ECs revealed that more than 95% of the cells expressed the specific EC marker CD31^27–30^, which was also used in the isolation procedure. This underlines the low number of contaminating non-endothelial cells. Moreover, the isolated cells were positive for VE-cadherin, a marker protein solely expressed by ECs^35–37^. These findings were confirmed by PCR analysis of ECs from four independent isolation procedures, which verified the expression of CD31, VE-cadherin and van Willebrand factor and thus, confirmed the endothelial origin of the isolated cells. The expression patterns of these genes were stable for 5 passages.

The functional properties of isolated endothelial cells were assessed by staining for N-cadherin, a major determinant of the vascular morphogenesis, proliferation and motility^38^. Diffuse cytoplasmatic staining of varying intensities was observed in the isolated nerve- derived ECs, a result which corresponds to the N-cadherin expression profiles found in HUVECs^37–40^. Moreover, labeling of the tight-junction associated protein ZO-1 revealed a high capacity of ECs to form tight junctions at the cellular contact sites. This is of physiological importance, since the tight junctions between endoneurial ECs are one of the important constituents of the BNB^3,4^. The capability of the isolated ECs to form capillary vessels was demonstrated when seeded on Matrigel under serum deprivation. Within 12 h, a dense network of EC-tubes was formed, making the isolated ECs a suitable tool for tube formation assays and further functional applications.

Given that basic research regarding the BNB and the vascularization of nerve lesions has mainly been assessed in animal models, our easy-to-follow protocol allows the use of primary human nerve-derived ECs to validate the results regarding the BNB and revascularization of nerve lesions in the human system. However, the use of human nerve derived ECs might be limited by the supply of human peripheral nerve samples, since autologous nerve transplantations are rare in many institutes and require ethical considerations. To circumvent the limited supply of tissue, we established a cryopreservation protocol for the isolated ECs. Prospectively, this would provide the possibility for the conduction of experiments independent of the current availability of sural nerve grafts and might also allow the transport of viable human nerve-derived ECs. However, since we yet have only assessed the cell viability following short-term cryopreservation at − 80 °C, experience regarding the long-term storage is pending.

In addition to the use in basic research, several clinical applications can be suggested: So far, HUVECs or rodent derived ECs were used for the experimental vascularization of hydrogels or artificial nerve grafts, prior to transplantation^41,42^. Therefore, a possible experimental application is the vascularization of artificial peripheral nerve grafts with autologous patient derived ECs, extracted for instance from neuroma biopsies. Another application could be the introduction of patient derived ECs into the suture site after in-vitro propagation. This could possibly improve the axonal crossing of the injury-site, as ECs were shown to mediate axonal guidance in rodents^5^. Thus, the staggered time course of axons crossing the injury site^43,44^ might be improved timewise.

## Conclusion

In conclusion, we developed a novel protocol for the efficient isolation of ECs from human peripheral nerves by means of immunomagnetic CD31 conjugated Dynabeads. The endothelial origin of the isolated cells was verified by immunocytology and revealed a purity > 95%. Thus, the isolated human nerve-derived ECs help to further improve basic research regarding the BNB and peripheral nerve regeneration in humans and could potentially be applied in vascularized artificial nerve grafts.

## Material and methods

### Ethics declaration

All experimental protocols were approved by the local ethics committee (“Ethik-Kommission der Carl von Ossietzky Universität Oldenburg” Drs. 49/2012). We confirm that all methods were performed in accordance with the relevant guidelines and regulations. An informed consent was obtained from all the patients.

### Coating of dynabeads with CD31 antibodies

For the preparation of Dynabeads, which is sufficient for 10 isolations of 5 cm sural nerve at a time, 50 µl streptavidin coated Dynabeads MyOne Streptavidin T1 (ø 1 µm, Invitrogen, Carlsbad, CA, USA) were deployed. The Dynabeads were washed three times in 1.5 ml reaction tubes using 1 ml isolation buffer (0.1% bovine serum albumin in 0.1 M physiological phosphate buffered saline (PBS), pH 7.4 consisting of 140 mM sodium chloride, 2.7 mM potassium chloride, 1.5 mM potassium dihydrogen phosphate and 8.1 mM disodium hydrogen phosphate) and the DynaMag-2 magnet (Invitrogen, Carlsbad, CA, USA) to remove sodium azide, which is contained as a preservative. Based on the Dynabeads-Streptavidin binding capacity of 200 µg biotinylated IgG for each ml of Dynabeads, 10 µg of biotinylated goat anti mouse IgG antibody (BA-9200, VectorLabs, Burlingame, CA, USA) were incubated with the washed Dynabeads in isolation buffer for 30 min at room temperature (RT). Slight rotation ensured proper orientation of the antibody. Following three washing steps in isolation buffer, 10 µg of anti-CD31 antibody (clone WM59, Biolegend, San Diego, CA, USA) in 40 µl isolation buffer were added under slight rotation for at least 1 h at RT (or 3 h at 4 °C), which resulted in binding of the anti-CD31 antibody to the Dynabeads via antibody-antibody interactions. Excess of primary antibodies was removed with three washing steps (isolation buffer) and the anti-CD31 conjugated Dynabeads were resuspended in 50 µl isolation buffer. Such a preparation can be stored at 4 °C for at least one week, when handling is done in a sterile environment.

### Human nerve preparation and digestion

Human sural nerve was removed aseptically during autologous nerve transplantations via nerve stripping and non-required remains were immediately stored in sterile physiological 0.1 M PBS, pH 7.4 at 4 °C. The nerve tissue was processed rapidly for high cell viability, although storage of the tissue is possible for up to 12 h at 4 °C in physiological PBS, pH 7.4. Excess of adipose and connective tissue was removed. To achieve the best yield, micro- and macrovascular cells were isolated by subsequent digestion of the whole nerve. For the isolation of pure microvascular endothelial cells, the nerve was dissipated in 3 cm parts and the fascicles were gently pulled out with micro-tweezers. A longitudinal incision was required for release in case fascicles were stuck in the epineurium. Subsequently, the whole nerve or isolated fascicles were chopped in small pieces with sterile scissors in 3 ml digestion media (M199, 20% FBS, 2.2 g/l sodium bicarbonate, 2.38 g/l HEPES, 100 000 units/l penicillin, 100 mg/l streptomycin, 0.1 mg/ml collagenase I and 0.1 mg/ml hyaluronidase; Sigma-Aldrich, St. Louis, MO, USA) in 4 cm cell culture plates (Thermo Fisher Scientific, Waltham, MA, USA) and digested for 12–15 h in a cell incubator at 37 °C with 5% CO_2_. The digested tissue was carefully triturated using a fire polished Pasteur pipette and pelleted at 800×*g* for 5 min.

### EC isolation with anti-CD31 conjugated Dynabeads

The pellet was resuspended in 100 µl isolation buffer containing 5 µl anti-CD31 conjugated Dynabeads and the suspension was incubated for 15 min at 4 °C with slight rotation. For positive isolation of ECs, 700 µl of isolation buffer was added, the reaction tubes were gently inverted and subsequently placed into the DynaMag-2 magnet for 5 min. The supernatant, containing non-endothelial cells, was removed, and Dynabeads with interacting ECs were subjected to three further washing steps in isolation buffer to remove contaminating cells. Finally, cells were resuspended in 1.5 ml EC-culture medium (M199, 20% FBS, 2.2 g/l sodium bicarbonate, 2.38 g/l HEPES, 100,000 units/l penicillin, 100 mg/l streptomycin, 100 µg/ml heparin, 50 µg/ml endothelial cell growth substrate; Sigma-Aldrich, St. Louis, MO, USA).

For verification of a successful binding of the anti-CD31 antibody to the Dynabeads as well as successful binding of the CD31-conjugated Dynabeads to endothelial cells, 5 µl of the isolated EC suspension can be retained and used as control: The CD31-antibody on the Dynabeads can be visualized by Alexa488 cross-linked goat anti-mouse IgG (Cat. No. A28175, Invitrogen, Karlsruhe, Germany). This results in green-fluorescent Dynabeads, bound to ECs, when observed with fluorescence microscopy. In case fluorescence of the ECs is observed, an excess of anti-CD31 antibody is present and the washing-steps during the anti-CD31-Dynabead conjugation procedure should be increased.

### Cultivation of ECs

ECs were seeded at a density of ~ 2000 cells/cm^2^ in gelatin-coated cell culture dishes or on coated coverslips (1% gelatin solution in ddH_2_O, 1 h at 37 °C). The cells were cultivated at 37 °C in the presence of 5% CO_2_ and the culture medium was exchanged 24 h after seeding and subsequently every three days. The heparin (Cat. No. H3149, Sigma-Aldrich, St. Louis, MO, USA) and endothelial cell growth substrate (Cat. No. E2759, Sigma-Aldrich, St. Louis, MO, USA) were added to the EC-culture medium immediately before use. When the cells reached 90% confluency, splitting in a 3:1 ratio was performed by using 0.25% trypsin and 1 mM EDTA in PBS (Biochrom, Berlin, Germany).

### Cryopreservation

For the cryopreservation of the isolated endothelial cells, the cells were frozen with an approximate rate of − 1 °C/min in freezing media containing 90% FBS and 10% dimethyl sulfoxide (DMSO, Cat. No. D5879, Sigma-Aldrich, St. Louis, MO, USA) in cryovials (Cat. Nr. T309-2A, Simport, Beloeil, CA) at − 80 °C. Freezing was carried out directly following the isolation procedure. For thawing, the cryovial was placed in a water bath at 37 °C. The thawed cells were immediately washed in EC-culture media and were seeded at a density of ~ 2000 cells/cm^2^.

### Cell viability

Following the nerve tissue digestion, the trypsinization-process during subculturing or cryopreservation, the cells viability was controlled by the trypan-blue viability assay. Therefore, 50 µl of 0.4% trypan-blue solution was added to 50 µl cell suspension and the number of unstained (viable) and stained (non-viable) cells were assessed in a Neubauer hemacytometer.

### Immunohistological staining

For immunohistological staining, endothelial cells grown on coverslips were rinsed in 0.1 M PBS, pH 7.4 and fixed with methanol (− 20 °C) for 10 min. After three washing steps in PBS at RT, unspecific binding-sites were blocked using 10% normal goat serum in 0.1 M PBS, pH 7.4. Incubation with the primary antibodies (see Table 1) was performed at RT for 3 h. Unbound primary antibodies were removed by three washing steps (3 × 5 min in PBS, pH 7.4) before the incubation with the corresponding Alexa488 conjugated secondary antibody (Cat. No. A28175, Invitrogen, Karlsruhe, Germany) was carried out at RT for 1 h. Finally, coverslips were rinsed (3 × 5 min in PBS, pH 7.4, 1 × in dd H_2_O) and mounted using Vectashield mounting medium containing DAPI (Invitrogen, Karlsruhe, Germany). Images were taken with a Leica DM6 widefield microscope and were deconvolved with Huygens Essential (Scientific Volume Imaging, Hilversum, Netherlands) according to the standard widefield settings. Brightness and contrast was adjusted using the scientific processing software Fiji^45^. All figures were prepared using Adobe Photoshop CS 6 (Adobe Inc., San Jose, CA, USA).

**Table 1:** Antibodies used for immunohistological staining.

Antibody	Clone	Manufacturer	Conc	RRID
Anti-CD31	WM59	Biolegend, San Diego, CA, USA	1:100	AB_314328
Anti-VE-Cad	BV9	Biolegend, San Diego, CA, USA	1:200	AB_10588267
Anti-N-Cad	3B9	Thermo Fisher Scientific, Waltham, MA, USA	1:100	AB_2313779
Anti-ZO-1	1A12	Thermo Fisher Scientific, Waltham, MA, USA	1:150	AB_2533147

The purity of the isolated ECs was assessed by the immunohistological analysis of CD31 immunoreactivity in confluent monolayers. The number of CD31 positive and negative cells were counted with the help of the “CellCounter” plugin in Fiji. Per each of four independent isolation procedures, five images were quantified.

### RNA isolation and PCR

RNA and Protein were isolated from confluent ECs of each well of a 6-well cell culture dish (each well ~ 10^6^ cells) using TRIzol (Thermo Fisher Scientific, Waltham, MA, USA) as specified in the data sheet. In brief, cells were rinsed with PBS (pH 7.4) and 1 ml of TRIzol was added to each well of a 6-well culture dish. Subsequently, cells were lysed by vigorous pipetting and the RNA was isolated from the lysate in a reaction tube by addition of 200 µl chloroform, followed by centrifugation and precipitation from the upper phase with isopropanol. The RNA pellet was washed with 100% ethanol for 5 min and resuspended in 50 µl RNAse free water (Qiagen, Hilden, Germany). The RNA was immediately subjected to DNAse I digestion (Invitrogen, Carlsbad, CA, USA) and transcribed with the Transcriptor High Fidelity cDNA Synthesis Kit (Roche, Mannheim, Germany) according to the data sheet. The PCR was performed with the TopTaq polymerase kit (Qiagen, Hilden, Germany) and primer pairs for CD31, VE-cadherin, von Willebrand factor (vWF) and glyceraldehyde 3-phosphate dehydrogenase (GAPDH) at 60 °C annealing temperature (see Table 2). The analysis was conducted in quadruplets using cDNA from four independent EC preparations derived from sural nerves of four different patients.

**Table 2:** Primers used for PCR. Sequences were obtained from PrimerBank^46^.

Primer	Sequence forward	Sequence reverse	Product
CD31	ACCGTGACGGAATCCTTCTCT	GCTGGACTCCACTTTGCAC	246 bp
vWF	AGCCTTGTGAAACTGAAGCAT	GCCCTGGTTGCCATTGTAATTC	237 bp
VE-Cad	GTTCACGCATCGGTTGTTCAA	CGCTTCCACCACGATCTCATA	238 bp
GAPDH	GGAGCGAGATCCCTCCAAAAT	GGCTGTTGTCATACTTCTCATGG	197 bp

### Tube formation assay

To support EC adhesion and EC-tube formation in cell culture, Matrigel (BD Biosciences, Franklin Lakes, NJ, USA) was used at a concentration of 5 mg/ml diluted with serum reduced culture medium (M199, 5% FBS, 2.2 g/l sodium bicarbonate, 2.38 g/l HEPES, , 100 000 units/l penicillin, 100 mg/l streptomycin, 100 µg/ml heparin, 50 µg/ml endothelial cell growth substrate; Sigma-Aldrich, St. Louis, MO, USA) Therefore, each well of a 24-well plate was covered with 100 µl Matrigel solution and kept at 4 °C, until further incubation was carried out to solidify Matrigel on the surface of the cell culture dish or cover slips at 37 °C for 1 h, just before ~ 5 × 10^4^ cells/well were seeded in serum reduced culture medium. The formation of ECs-tubes was assessed after 12 h.

## Supplementary Information

Supplementary Information.

## Data Availability

The data generated during and/or analyzed during the current study are available from the corresponding author on reasonable request.
