# Supplementary material for: Rapid and efficient immunomagnetic isolation of endothelial cells from human peripheral nerves
Source: Sci Rep. 2021 Jan 21;11:1951. doi: 10.1038/s41598-021-81361-x (PMC7820485; doi:10.1038/s41598-021-81361-x)
Supplement: Supplementary file 1 — Supplementary Information. [file 41598_2021_81361_MOESM1_ESM.docx]

**Supplementary Information**

**Title: Rapid and efficient immunomagnetic isolation of endothelial cells from human peripheral nerves**

**Authors: Patrick Dömer, Janine Kayal, Ulrike Janssen-Bienhold, Bettina Kewitz, Thomas Kretschmer, Christian Heinen**


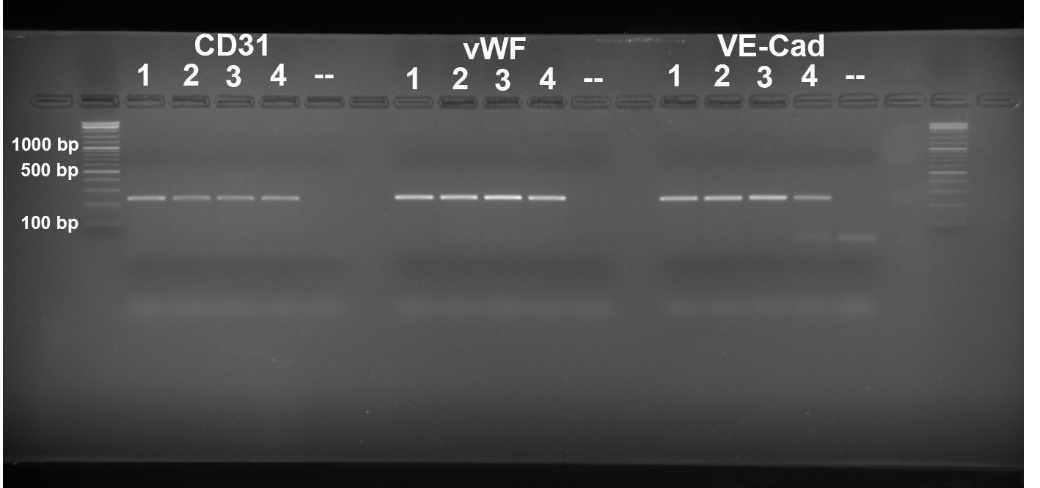

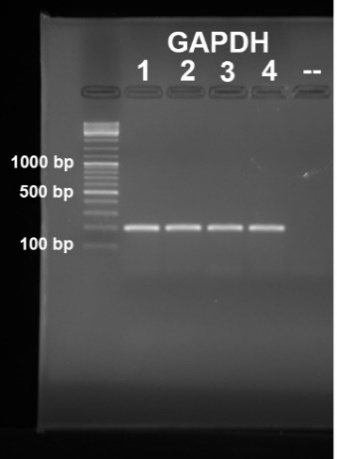


**Supplementary Figure S1: Uncropped gels confirming mRNA-Expression of the endothelial cell specific markers CD31, von Willebrand factor and VE-Cadherin via RT-PCR in four independent EC isolations (1-4).** Equal amounts of cDNA were used as verified by the GAPDH positive control. Omission of the template cDNA (--) revealed no signal.


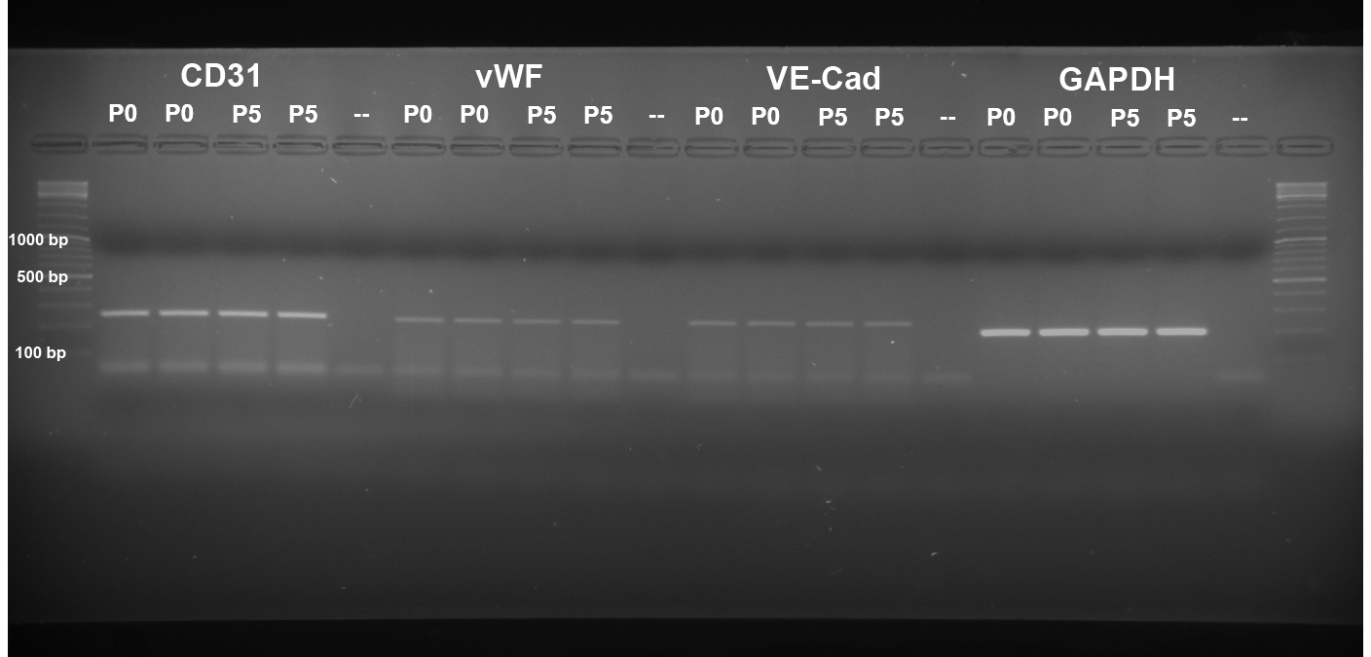


**Supplementary Figure S2: Uncropped gels confirming unchanged mRNA-Expression of the endothelial cell specific markers CD31, von Willebrand factor and VE-Cadherin from passage 0 (isolation) to passage 5 via RT-PCR.** Equal amounts of cDNA were used as verified by the GAPDH positive control. Omission of the template cDNA (--) revealed no signal.
